# Association of endothelin-1 and matrix metallopeptidase-9 with metabolic syndrome in middle-aged and older adults

**DOI:** 10.1186/s13098-015-0108-2

**Published:** 2015-12-10

**Authors:** A. P. Yu, B. T. Tam, W. Y. Yau, K. S. Chan, S. S. Yu, T. L. Chung, P. M. Siu

**Affiliations:** Department of Health Technology and Informatics, Faculty of Health and Social Sciences, The Hong Kong Polytechnic University, Hung Hom, Kowloon, Hong Kong, China

**Keywords:** Metabolic syndrome, Cardiovascular disease, Diabetes mellitus, Cancer, ET-1, MMP-9

## Abstract

**Background:**

Metabolic syndrome (MetS) contains a cluster of cardiovascular risk factors. People with MetS are more susceptible to cardiovascular disease, diabetes mellitus, and cancer. Endothelin-1 (ET-1) and matrix metallopeptidase-9 (MMP-9) have been implicated in the development of cardiovascular diseases, diabetes mellitus and cancers. This cross-sectional study aimed to examine the association of ET-1 and MMP-9 with MetS in middle-aged and older Hong Kong Chinese adults.

**Methods:**

149 adults aged 50 to 92 (n = 75 for non-MetS group and n = 74 for MetS group) were examined. All subjects were screened for MetS according to the diagnostic guideline of the United States National Cholesterol Education Program (NCEP) Expert Panel Adult Treatment Panel (ATP) III criteria. Serum levels of ET-1 and MMP-9 were measured. Independent *t* test was used to detect differences between non-MetS and MetS groups and between subjects with or without certain metabolic abnormality. The association of the serum concentration of MMP-9 and ET-1 with MetS parameters were examined by Pearson’s correlation analysis.

**Results:**

Serum level of ET-1 is higher in MetS-positive subjects and in subjects with high blood pressure, elevated fasting blood glucose, and central obesity. The serum concentration of MMP-9 is higher in subjects positively diagnosed with MetS and subjects with high blood pressure, elevated fasting blood glucose, low blood high-density lipoprotein-cholesterol (HDL-C), high blood triglycerides, and central obesity. Correlation analyses revealed that serum concentration of ET-1 is positively correlated to systolic blood pressure, waist circumference, fasting blood glucose, and age whereas it is negatively correlated to HDL-C. MMP-9 is positively correlated to systolic blood pressure, waist circumference, fasting blood glucose, and age whereas it is negatively correlated to HDL-C.

**Conclusion:**

Serum ET-1 is higher in subjects with hypertension, hyperglycemia, central obesity or MetS. Serum MMP-9 is higher in subjects diagnosed with MetS or having either one of the MetS parameters. Both circulating levels of ET-1 and MMP-9 are correlated to systolic blood pressure, waist circumference, fasting blood glucose, HDL-C, and age. Further research is needed to fully dissect the role of ET-1 and MMP-9 in the development of cancers, diabetes and cardiovascular disease in relation to MetS.

## Background

Metabolic syndrome (MetS) is regarded as a sub-health status that contains a clustering of cardiovascular risk factors, including high blood pressure, central obesity, insulin resistance, hypertriglyceridemia and dyslipidemia [1–4]. People diagnosed with MetS are known to be more susceptible to develop diabetes mellitus [5], cardiac vascular diseases [6], and cancers [7, 8]. It has been demonstrated that the circulating levels of ET-1 and MMP-9 are higher in patients with diabetes mellitus [9, 10], cardiovascular diseases [11, 12], and cancers [13, 14].

ET-1 is a 21 amino acids-long vasoconstrictor, mitogen and pro-inflammatory peptide which is mainly produced in endothelial cells in small amount under normal physiological condition [15, 16]. However, its abundance is highly increased during pathological conditions due to the stimulated production in different types of cell including endothelial cells, cardiac myocytes, smooth muscle cells and inflammatory cells [11, 17, 18]. Studies have revealed that ET-1 also contributes to several pathological events including inflammation [19–22], fibrosis [23], cardiac and vascular hypertrophy [24] and proliferation of vascular smooth muscle cells and fibroblasts [24]. The upregulation of ET-1 is regarded as an important pathogenic factor of the development of cardiovascular diseases [11]. Removal of ET-1 in cardiomyocytes has been demonstrated to ameliorate the cardiac remodeling caused by Trypanosoma cruzi-induced heart disease [25]. Elevated plasma ET-1 level is also observed in patients of diabetes mellitus [9] and cardiovascular diseases [11] while ET-1 has been suggested to be related to the promotion of cancer development by modulating mitosis, angiogenesis and apoptosis [13].

Matrix metalloproteinases (MMP) is a protein family of zine-dependent endopeptidases that regulate tissue remodeling under both normal physiological and pathological conditions [12]. Among them, MMP-9, also known as gelatinase B or 92-kDa type IV collagenase, is responsible for the degradation of extracellular matrix [12]. Abundance of MMP-9 is increased in several cardiovascular diseases including hypertension [26], atherosclerosis [27] and myocardial infarction [28]. Elevation of MMP-9 abundance may initiate or exacerbate the pathogenesis due to immune response stimulated by its proteolytic property [12, 28]. It has been demonstrated that the circulating level of MMP-9 is increased under hyperglycemia condition [29]. General deletion or target deletion of MMP-9 has been shown to alleviate the pathological conditions caused by downregulation of ET-1, hence prevented enlargement of left ventricle under myocardial infarction by reducing the inflammatory response and improving left ventricle remodeling in the permanent occlusion model of myocardial infarction [30]. These results support the genetic studies showing that MMP-9 plays an important role in cardiovascular diseases [12, 30]. MMP-9 has also been shown to be involved in angiogenesis [31] and metastasis of certain cancers [32].

ET-1 and MMP-9 are both endothelial biomarkers that may contribute to the development of endothelial dysfunction and are involved in cardiac remodeling and the development of cardiovascular diseases [33]. Notably, one of the underlying causes of diabetic angiopathy, cardiovascular diseases, diabetes mellitus and its complications was thought to be related to endothelial dysfunction [34]. It has been previously demonstrated by a population-based study that the increase in circulating endothelial biomarkers, such as ET-1 and MMP-9, can be used to evaluate the endothelial function and predict the risk of diabetes [35]. The change in these endothelial biomarkers may provide clues to evaluate the high susceptibility to cardiovascular diseases, diabetes mellitus and cancers for people with MetS. This cross-sectional study aimed to examine the association of ET-1 and MMP-9 with MetS in middle-aged and older Hong Kong Chinese adults.

## Methods

Middle-aged and older Hong Kong Chinese adults with age ranged from 50 to 92 including 75 MetS negative and 74 MetS positive subjects were voluntarily recruited. Participants with dementia or mental disorders, severe or acute cardiovascular diseases, post-stroke, neuromusculoskeletal illness, acute medical illness, symptomatic heart or lung diseases, severe rheumatoid arthritis, osteoarthritis or pulmonary illness and participants who were immobile, smoker or under treatment for metabolic abnormalities were excluded in this study. Subjects were informed about the potential risks and benefits of their participation, and written informed consent was obtained on a voluntary basis before the study began. All the experimental procedures received human research ethics approval from The Hong Kong Polytechnic University (HSEARS20150116001 and HSEARS20150116002). All subjects were screened for MetS according to the diagnostic guideline of the United States National Cholesterol Education Program (NCEP) Expert Panel Adult Treatment Panel (ATP) III criteria. Individuals diagnosed with MetS have more than two of following characteristics: (1) central obesity (waist circumference exceeds 90 or 80 cm for Asian male and female, respectively), (2) hypertension (systolic pressure equals or exceed 130 mmHg or diastolic pressure equals or exceeds 85 mmHg), (3) elevated blood glucose (fasting glucose level equals or exceeds 5.5 mmol/L [100 mg/dL]), (4) elevated plasma triglycerides (level equals or exceeds 1.70 mmol/L [150 mg/dL]), and (5) low level of high-density lipoprotein-cholesterol (HDL-C; level equals or is less than 40 mg/dL for male and 50 mg/dL for female) are regarded as MetS positive.

### Measurement of MetS diagnostic parameters and the circulating endothelial biomarkers

MetS diagnostic parameters including blood pressure, waist circumference, fasting blood glucose, blood triglycerides and blood HDL-C were measured. Systolic blood pressure, diastolic blood pressure and waist circumference were measured by trained research personnel. Blood pressure measurement was determined on the right arm after 5-min seated rest using an electronic blood pressure monitor (Accutorr Plus, Datascope). Systolic and diastolic blood pressure was obtained over the brachial artery region with the arm supported at heart level using appropriate sized cuff. The average of two measurements taken with a 1-min interval between them was recorded for analysis. Waist circumference was measured midway between the lowest rib and the superior border of the iliac crest using an inelastic measuring tape on the bare skin and recorded to the nearest 0.1 cm. The tape was snugged horizontally around the abdomen passing across the navel without causing compression on the skin. Measurement was performed at the end of normal expiration. Fasting venous blood samples were collected after a minimum 10 h fast by certified phlebotomists for biochemical measurements. Fasting blood glucose, blood triglycerides, and blood HDL-cholesterol concentrations were measured by an accredited medical laboratory by commercial test kit methods using an automatic clinical chemistry analyser (Architect CI8200, Abbott Diagnostics). The serum ET-1 level was assayed by a commercially available chemiluminescent enzyme-linked immunoassay kit (QuantiGlo® Human Endothelin-1 Chemiluminescent Immunoassay, R&D Systems, USA) and the serum MMP-9 level was detected by Biotrak™ MMP-9 activity assay (GE Healthcare) according to the protocols provided by manufacturers.

### Statistical analysis

Subjects were grouped according to different criteria including their MetS status and the presence of each cardio-metabolic abnormality. Data are expressed as mean ± standard deviation. Normalities of the data were examined. The statistical differences of subjects’ gender ratios with certain cardio-metabolic abnormality were assessed by Chi square test. The differences between two groups were detected by independent *t* test. Pearson’s correlation analysis was performed to examine the correlation between the serum concentration of MMP-9 and ET-1 to the MetS parameters. All statistical analyses were performed using the Statistical Package for the Social Sciences (SPSS) version 21 for Windows. Statistical significance was accepted at *p* < 0.05.

## Results

One hundred and forty-nine adults (n = 75 for MetS negative subjects and n = 74 for MetS positive subjects) were included in this cross-sectional study. The gender ratio of female-to-male was 83-to-66. The number of subjects, gender ratio and age of subjects with certain cardio-metabolic abnormality are summarized in Table 1. It is observed that the age of the subjects with central obesity (P = 0.038) and with dyslipidemia (P = 0.001) are significantly higher than those without in the present study. The gender ratios of subjects with certain cardio-metabolic abnormality are not significantly different (Table 1).

**Table 1:** Background information of subjects

	General information of recruited subjects	
Age	64.6 ± 9.6 years (Range: 50–92)	
Gender ratio (F:M)	83:66	

	Information of subjects positively diagnosed with			Information of subjects negatively diagnosed with			P-value of age	P-value of gender ratio (Chi square test)
	No. of subject	Age	Gender ratio (F:M)	No. of subject	Age	Gender ratio (F:M)		
Metabolic Syndrome	75	62.29 ± 7.17	41:34	74	67.03 ± 11.09	42:32	0.002	0.797
Hypertension	61	63.03 ± 7.45	37:24	88	65.76 ± 10.73	46:42	0.069	0.331
Central Obesity	69	62.94 ± 7.02	36:33	80	66.11 ± 11.19	47:33	0.038	0.420
Hypertriglyceridemia	101	64.18 ± 7.80	54:47	48	65.63 ± 11.12	29:19	0.431	0.425
Dyslipidemia (low HDL-C)	94	61.34 ± 7.08	50:44	55	70.29 ± 10.70	33:22	0.001	0.419
Hyperglycemia	103	64.29 ± 8.91	60:43	46	65.43 ± 11.03	23:23	0.538	0.349

P = 0.001

**Fig. 1:**
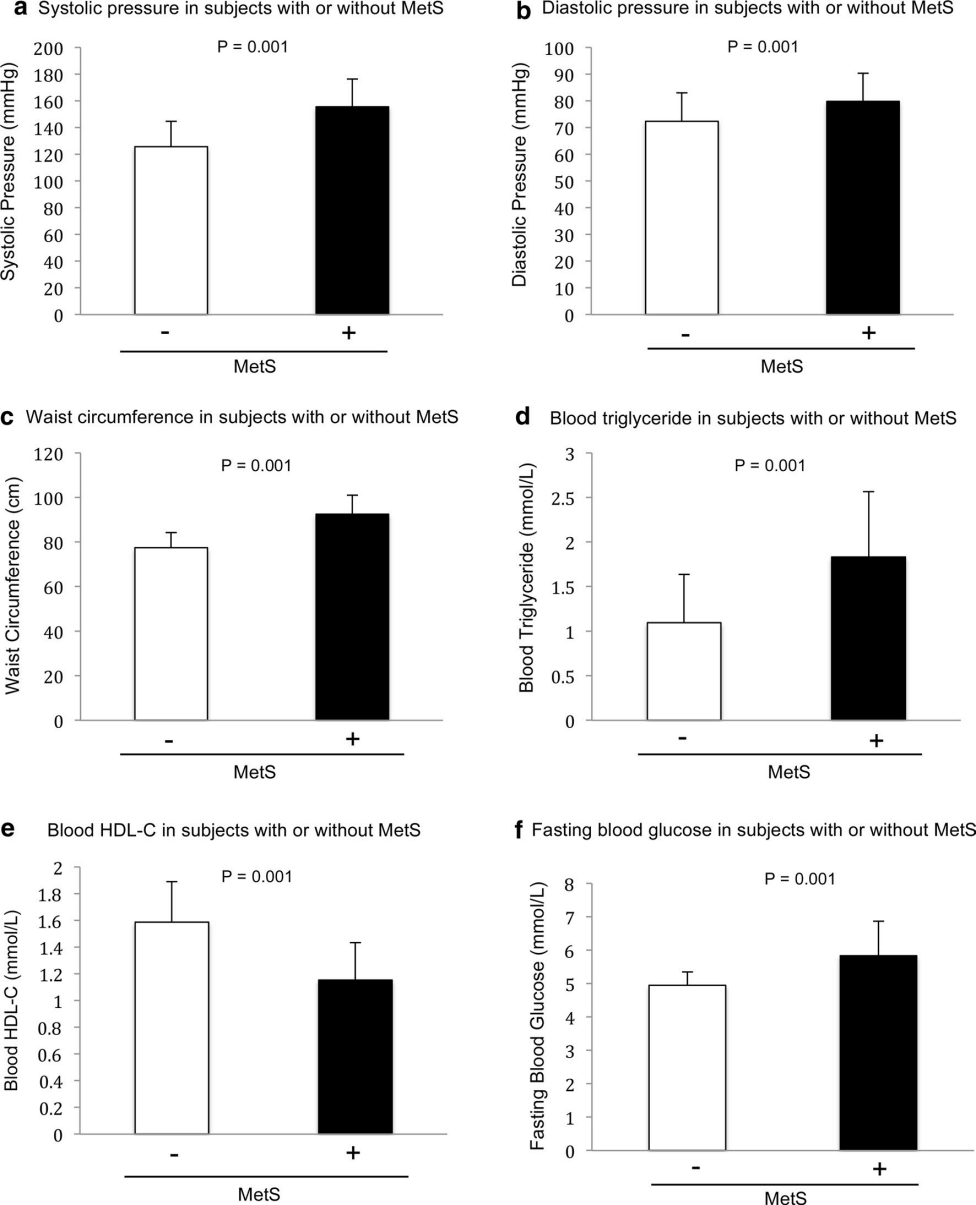
MetS parameters in subjects with or without MetS and metabolic abnormalities. **a** Systolic pressure in subjects with or without MetS. **b** Diastolic pressure in subjects with or without MetS. **c** Waist circumference in subjects with or without MetS. **d** Blood triglycerides in subjects with or without MetS. **e** HDL-C in subjects with or without MetS. **f** Fasting blood glucose in subjects with or without MetS. Data are expressed as mean ± standard deviation. The differences between two groups were detected by independent t-test. Statistical significance was accepted at *p* < 0.05

The systolic blood pressure (Fig. 1a), diastolic pressure (Fig. 1b), waist circumference (Fig. 1c), blood triglycerides (Fig. 1d) and fasting blood glucose (Fig. 1f) are all significantly higher in subjects with MetS compared to MetS-negative subjects. The blood HDL-C in MetS positive subjects is significantly lower when compared to those without MetS (Fig. 1e).

**Fig. 2:**
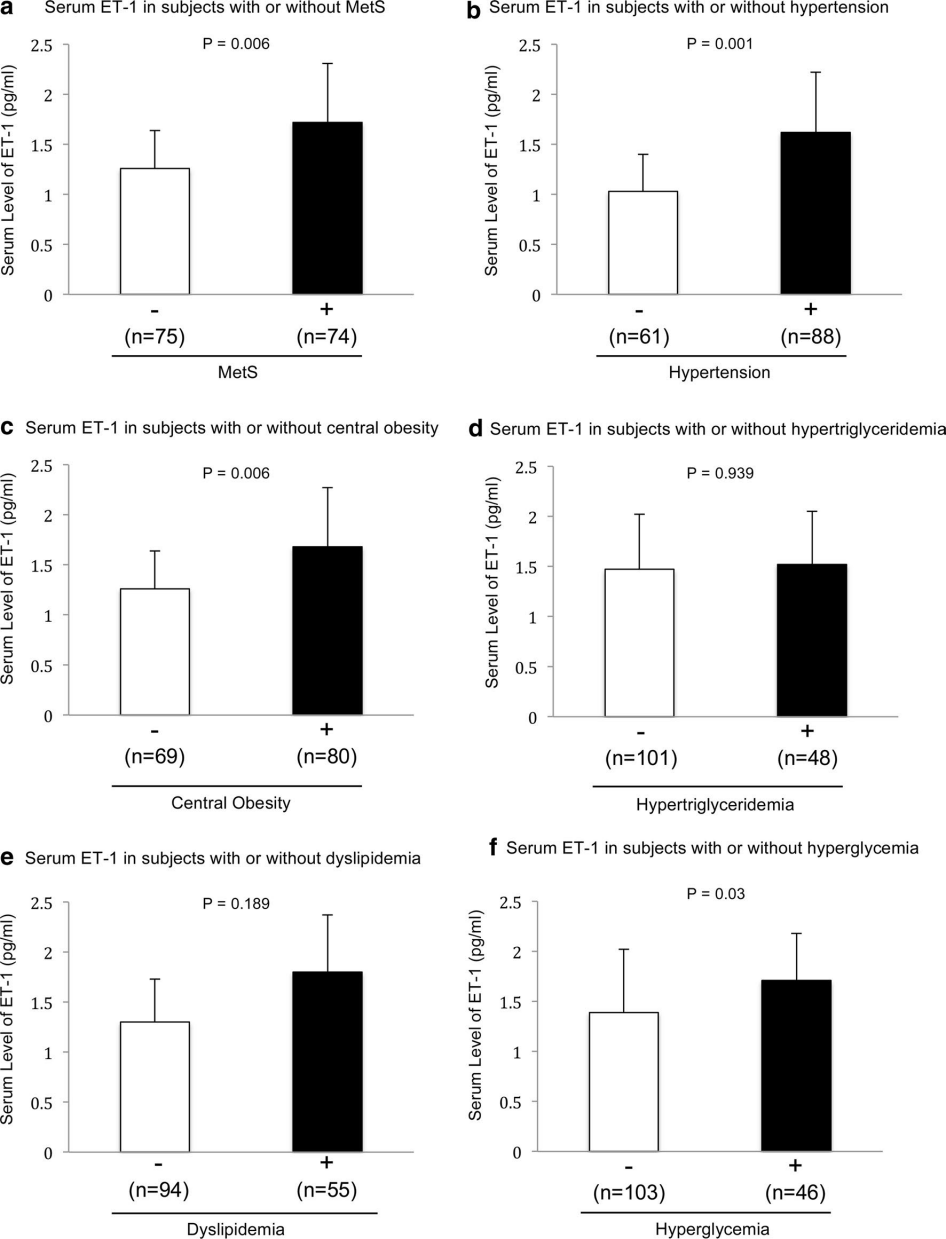
Serum level of ET-1 in subjects with or without MetS and metabolic abnormalities. **a** Serum level of ET-1 in subjects with or without MetS. **b** Serum level of ET-1 in subjects with or without hypertension. **c** Serum level of ET-1 in subjects with or without central obesity. **d** Serum level of ET-1 in subjects with or without hypertriglyceridemia. **e** Serum level of ET-1 in subjects with or without dyslipidemia (low HDL-C). **f** Serum level of ET-1 in subjects with or without hyperglycemia. Data are expressed as mean ± standard deviation. The differences between two groups were detected by independent t-test. Statistical significance was accepted at *p* < 0.05

**Fig. 3:**
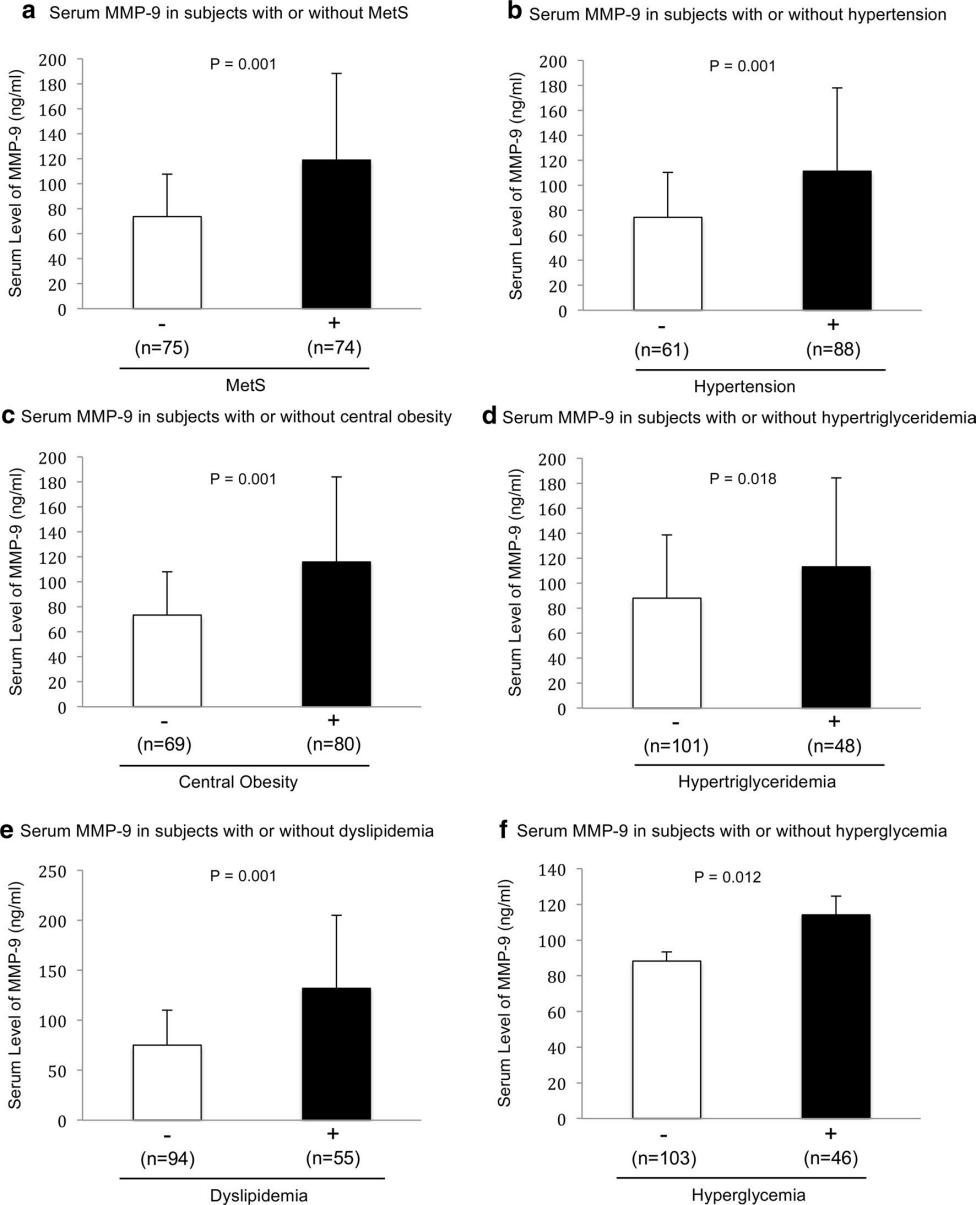
Serum level of MMP-9 in subjects with or without MetS and metabolic abnormalities. **a** Serum level of MMP-9 in subjects with or without MetS. **b** Serum level of MMP-9 in subjects with or without hypertension. **c** Serum level of MMP-9 in subjects with or without central obesity. **d** Serum level of MMP-9 in subjects with or without hypertriglyceridemia. **e** Serum level of MMP-9 in subjects with or without dyslipidemia (low HDL-C). **f** Serum level of MMP-9 in subjects with or without hyperglycemia. Data are expressed as mean ± standard deviation. The differences between two groups were detected by independent t-test. Statistical significance was accepted at *p* < 0.05

The serum ET-1 concentration is significantly higher in the MetS-positive subjects when compared to the MetS-negative subjects (P = 0.006) (Fig. 2a). The serum concentration of ET-1 in subjects with hypertension (P = 0.001) (Fig. 2b), central obesity (P = 0.006) (Fig. 2c), and high fasting blood glucose (P = 0.03) (Fig. 2f) are significantly higher than those without. The serum concentration of MMP-9 in subjects diagnosed with MetS (P = 0.001) (Fig. 3a), hypertension (P = 0.001) (Fig. 3b), central obesity (P = 0.001) (Fig. 3c), high blood triglycerides (P = 0.018) (Fig. 3d), dyslipidemia or low HDL-C (P = 0.001) (Fig. 3e), and high fasting blood glucose (P = 0.012) (Fig. 3f) are significantly higher than subjects negatively diagnosed with those parameters. Neither the circulating level of ET-1 nor MMP-9 has shown a significant difference between male and female (data not shown).

**Fig. 4:**
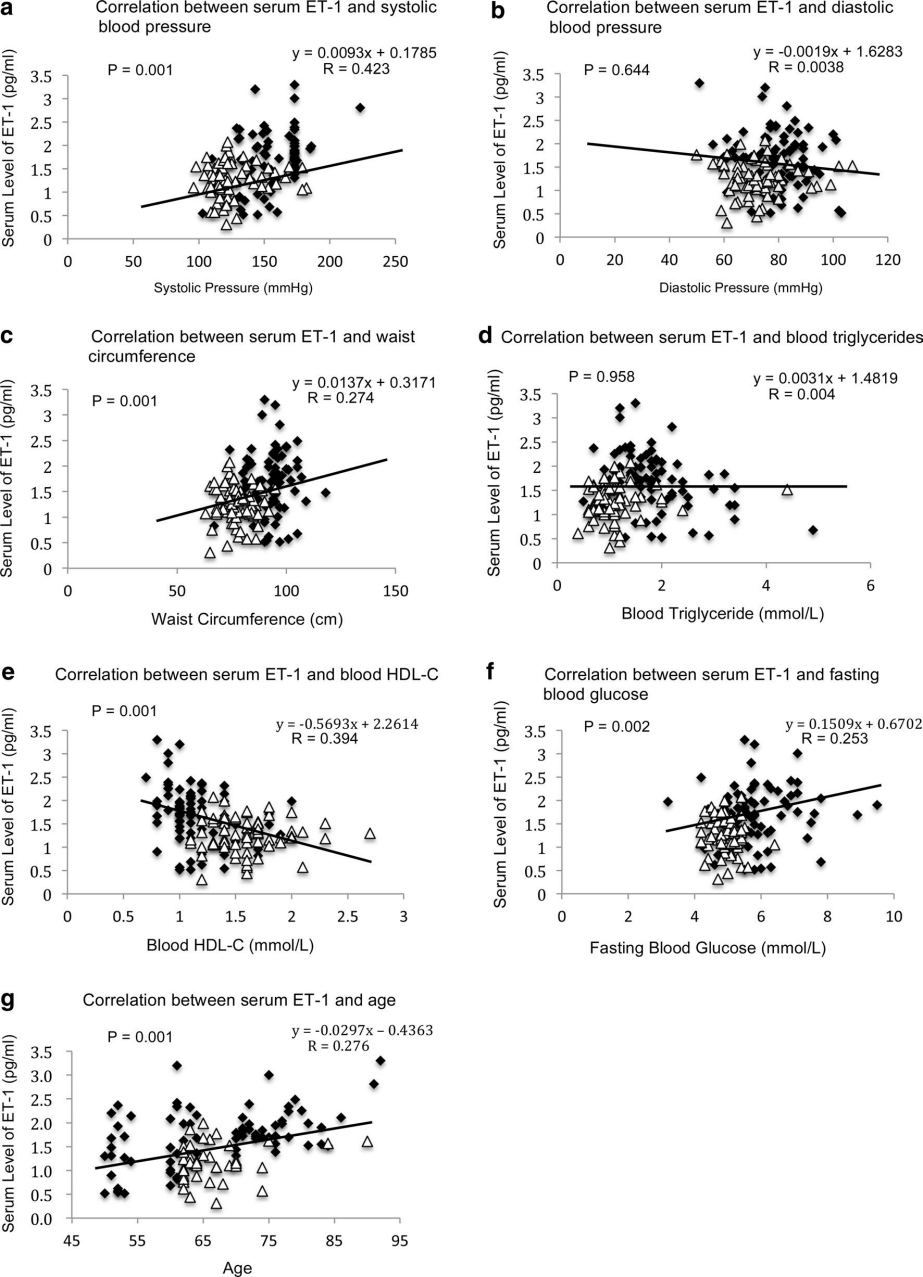
Correlation analysis of serum level of ET-1 to parameters of metabolic syndrome. **a** Correlation between serum level of ET-1 and systolic blood pressure. **b** Correlation between serum level of ET-1 and diastolic blood pressure. **c** Correlation between serum level of ET-1 and waist circumference. **d** Correlation between serum level of ET-1 and blood triglycerides. **e** Correlation between serum level of ET-1 and blood HDL-C. **f** Correlation between serum level of ET-1 and fasting blood glucose. **g** Correlation between serum level of ET-1 and age. Pearson’s correlation analysis was performed. Statistical significance was accepted at *p* < 0.05. *Black diamond* refers to the data of MetS-positive subjects. *Open triangle* refers to the data of MetS-negative subjects

**Fig. 5:**
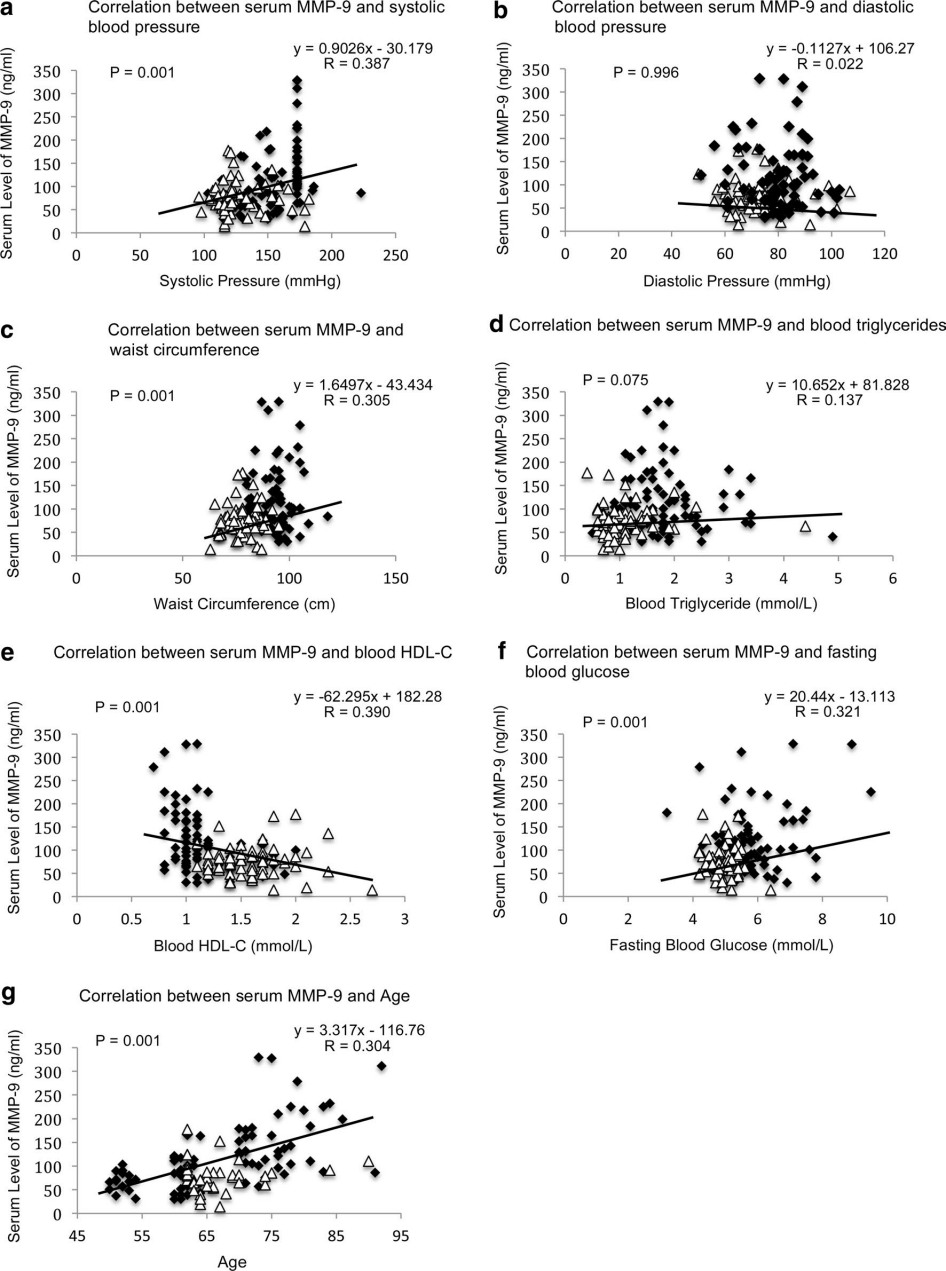
Correlation analysis of serum level of MMP-9 to parameters of metabolic syndrome**. a** Correlation between serum level of MMP-9 and systolic blood pressure. **b** Correlation between serum level of MMP-9 and diastolic blood pressure. **c** Correlation between serum level of MMP-9 and waist circumference. **d** Correlation between serum level of MMP-9 and blood triglycerides. **e** Correlation between serum level of MMP-9 and blood HDL-C. **f** Correlation between serum level of MMP-9 and fasting blood glucose. **g** Correlation between serum level of MMP-9 and age. Pearson’s correlation analysis was performed. Statistical significance was accepted at *p* < 0.05. *Black diamond* refers to the data of MetS-positive subjects. *Open triangle* refers to the data of MetS-negative subjects

The correlation analyses revealed that the concentration of ET-1 was positively correlated to systolic blood pressure (P = 0.001, R = 0.423) (Fig. 4a), waist circumference (P = 0.001, R = 0.274) (Fig. 4c), fasting blood glucose (P = 0.002, R = 0.253) (Fig. 4f) and age (P = 0.001, R = 0.276) (Fig. 4g) whereas negatively correlated to HDL-C (P = 0.001, R = 0.394) (Fig. 4e). Similar pattern was observed in the correlation analyses of the serum concentration of MMP-9. The level of MMP-9 was positively correlated to systolic blood pressure (P = 0.001, R = 0.387) (Fig. 5a), waist circumference (P = 0.001, R = 0.305) (Fig. 5c), fasting blood glucose (P = 0.001, R = 0.321) (Fig. 5f), age (P = 0.001, R = 0.304) (Fig. 5g) and negatively correlated to HDL-C (P = 0.001, R = 0.390) (Fig. 5e).

## Discussion

MetS is a cluster of cardiovascular risk factors and people with MetS are known to be more susceptible to develop cardiovascular diseases, diabetes mellitus and common cancers [1–4]. A meta-analysis has revealed that the incident rate of people with metabolic syndrome of having cardiovascular disease, coronary heart disease and stroke is 50 % higher than healthy control [6]. Elevation of circulating levels of both ET-1 and MMP-9 are observed in patients with cardiovascular diseases, diabetes mellitus and cancers. Endothelium has been indicated as the primary site of dysfunction in cardiovascular disease while endothelial dysfunction has been hypothesized as one of the underlying causes for diabetes mellitus and its complications [34, 36]. Elevations of ET-1 and MMP-9 in the circulating system have been suggested to be associated with the development of endothelial dysfunction [33, 37]. The changes in circulating levels of ET-1 and MMP-9 might be associated with the possible mechanisms such as endothelial dysfunction that might help to explain why people with MetS have a higher chance of developing cardiovascular diseases, diabetes mellitus and cancers.

### Elevated circulating levels of ET-1 and MMP-9 in people with MetS might be associated with the increased risk of developing cardiovascular diseases and diabetes mellitus

ET-1 is a vasoconstrictor responsible for reducing heart rate, cardiac output and increasing mean arterial blood pressure via ET receptor [11]. Contribution of the elevation of plasma ET-1 to the development of clinical hypertension, vascular dysfunction and cardiac vascular diseases have been well recognized [11]. Yang and colleagues have generated a line of conditional overexpressing transgenic mice by inserting a human ET-1 cDNA to the downstream of alpha-MHC promoter to investigate the consequence of ET-1 overexpression in heart [21]. They observed increase in left ventricular endocardial circumference together with macrophages and T-cells infiltration, suggesting that upregulation of ET-1 may cause inflammation and dilated cardiomyopathy [21]. The normal range of the circulating ET-1 is 1.41 ± 0.50 pg/ml [38], which is consistent with the results reported in the present study. Subjects with MetS are demonstrated to have a higher circulating level of ET-1 when compared to healthy control, which might contribute to the higher risk of cardiovascular diseases in people with MetS. Our data also show that subjects with either elevated fasting blood glucose, central obesity or hypertension have higher serum level of ET-1. The serum concentration of ET-1 is found to be significantly correlated to systolic blood pressure, waist circumference, blood HDL-C and fasting blood glucose. The plasma ET-1 level has been reported to be elevated in patients with diabetes mellitus [9]. It has been demonstrated that hyperglycemia upregulates ET-1 in endothelial cells by mediating through AMPK-C/EBP signaling pathway [39]. Parrinello and colleagues compared the plasma concentrations of ET-1 in people with central obese normotensive and central obese hypertensive to lean normotensive people [40]. They have observed that both subjects with central obese normotensive and central obese hypertensive have significantly higher plasma concentration of ET-1 when compared to lean normotensive subjects [40]. Our results are in line with these findings that significantly higher abundance of ET-1 is found in subjects with central obesity. Leung and colleagues have demonstrated that chronic overexpression of ET-1 in endothelial cells results in elevation of blood pressure in a transgenic mice model [41]. In addition, a human study observed that both hypertensive and non-hypertensive subjects with phaeochromocytoma have a significantly higher level of ET-1 when compared to those with essential hypertension and healthy control [42]. Notably, association between the highest ET-1 level and the presence of hypertension has been shown in those subjects with phaeochromocytoma, suggesting that ET-1 might play a role in the development of clinical hypertension [42]. In the present study, it is observed that subjects with hypertension have a higher serum level of ET-1 when compared to non-hypertensive subjects. Hypertension has been shown to be highly associated with obesity [43, 44]. It is speculated that the obesity-associated increase in ET-1 might, at least partly, contribute to the development of hypertension and results in a high association between obesity and hypertension. Similarly, hyperglycemia-induced elevation of ET-1 might lead to changes in vascular structure and vascular function hence results in hypertension. The obesity-associated increase in ET-1 and hyperglycemia-induced elevation of ET-1 might partly explain the high association between hypertension and obesity as well as between hypertension and hyperglycemia.

Matrix metalloproteinases are the family of proteins responsible for cleaving the structural elements of the extracellular matrix (ECM) during physiological and pathological ECM remodeling. MMP-9, also known as gelatinase B or 92-kDa type IV collagenase belongs to the gelatinase subgroup of the matrix metalloproteinases family [12]. The circulating MMP-9 in healthy people normally ranges from 30 ng/ml to 537 ng/ml [45]. In the present study, serum levels of MMP-9 in subjects with or without MetS are fallen in the normal range. However, the serum level of MMP-9 is significantly elevated in subjects with MetS and subjects with either one of the cardio-metabolic abnormalities (i.e., central obesity, low HDL-C, hypertension, elevated fasting blood glucose and high blood triglycerides), suggesting that any one of the cardio-metabolic abnormalities might be associated with the elevation of circulating MMP-9. This perspective is also partly supported by our correlation analyses, which demonstrate the positive correlation between serum MMP-9 and systolic pressure, waist circumference and fasting blood glucose, as well as a negative correlation between serum MMP-9 and HDL-C. Of note, our results are in line with the literatures reporting that the circulating MMP-9 level is higher in patients with dyslipidemia [46], hypertension [26, 47], hyperglycemia [48] and central obesity [49]. It has also been previously demonstrated that MMP-9 level is correlated with blood triglycerides level [50] while our data show that the serum MMP-9 level is higher in people with hypertriglyceridemia.

### Plenty of studies have demonstrated the relationship between MMP-9 and diabetes mellitus

It has been demonstrated that the activities of MMP-9 in plasma and vascular cells are also higher in rodent models of diabetes mellitus [48]. Previous studies have demonstrated that MMP-9 level is significantly increased in patients under diabetic condition [10, 51]. Patients suffering from type 1 diabetes mellitus have been demonstrated to have significantly higher abundance of circulating MMP-9 [10]. MMP-9 activity has also been shown to be significantly higher in patients with type 2 diabetes mellitus [48, 52]. Notably, it is observed in the present study that subjects with fasting blood glucose equals or exceed 5.5 mmol/L have a significant higher level of serum MMP-9 level compared to subjects without hyperglycemia, suggesting that the upregulation of MMP-9 occurs before the fasting blood glucose level has reached to clinical diabetic level. MMP-9 is associated with several pathological changes of diabetes mellitus including diabetic retinopathy [52], diabetic nephropathy [10] and diabetic microvascular complications due to its contribution in microvascular remodeling [53]. Upregulation of MMP-9 has been observed during the development of diabetic microvascular complications [53]. The high abundance of MMP-9 is also associated with the recruitment of leukocytes to lesions and fibrosis of the microvascular wall [54, 55]. The activation of MMP-9 has been demonstrated to contribute to the development of retinopathy by increasing vascular permeability and enhancing apoptosis of retinal capillary cells [52]. Suppression of hyperglycemia-induced activation of MMP-9 can ameliorate apoptosis of retinal capillary cells [45]. Genetic variances on MMP-9 gene are demonstrated to be associated with the risk of developing diabetic microvascular complications. A study of meta-analysis has revealed that mutation of the promoter of MMP-9 gene decreases the risk of diabetic nephropathy [53]. The involvement of MMP-9 in the early hypertensive remodeling has been demonstrated [26] while MMP-9 has been suggested as a biomarker for acute coronary syndrome [56]. MMP-9 has also been suggested to be involved in plaque formation, destabilization and rupture [27, 57]. High abundance of MMP-9 has been observed in an animal model of hypertension [58] and clinically in women with gestational hypertension [59]. It has been demonstrated in the Framingham Offspring Study that high MMP-9 level is related to higher risk of blood pressure progression [60]. The incidence of coronary heart disease has been reported to be associated with the serum level of MMP-9 [61]. Interestingly, a study in middle-aged population revealed that the circulating level of MMP-9 is associated with psychosocial instruments covered depression, hostile affect, cynicism and sense of coherence, suggesting that the MMP-9 level might not only associated with the traditional risk factors but also with the psychosocial risk factors of cardiovascular diseases [62]. In this study, subjects with MetS or subjects with either one of metabolic abnormalities are observed to have significantly higher abundance of MMP-9 in the blood. These data support the speculation that hyperglycemia-induced upregulation of MMP-9 might be one of the reasons for diabetic patients having two- to four-fold increase in risk of having cardiovascular diseases when compared to non-diabetic people [63].

Notably, it has been observed that both ET-1 and MMP-9 are positively correlated with age but not gender. The age of subjects with central obesity and dyslipidemia are significantly higher than those without central obesity and dyslipidemia in this study. It has been previously reported that the prevalence of MetS increases with age [64, 65]. It has been demonstrated in the present study that not only the incidence rates of those cardio-metabolic abnormalities [64, 65], but also these two endothelial biomarkers are associated with age. The increases in these endothelial biomarkers with advancing age might partly explain the increase in the incidence rates of cardiovascular diseases [66] and diabetes mellitus [67] in elderly.

### Elevated circulating levels of ET-1 and MMP-9 in people with MetS might be related to higher risk of cancer development

There are literatures supporting that people with MetS have increased risk of developing common cancers due to the association between the MetS components and etiology or progression of certain cancers [68]. Cancer patients with obesity tend to manifest more localized tumors, earlier relapse and lower survival rate [69]. Studies have shown that obesity is linked to cancer of the gastricardia, cholangiocarcinoma, esophageal adenocarcinoma [70], multiple myeloma and large B cell lymphoma in men [71] and breast cancer in women [70]. In addition, the association of dyslipidemia/hyperglycemia and cancers has been reported. The incidences of lung cancer [70], non-hodgkin lymphoma [71] and breast cancer [72, 73] are associated with low circulating level of HDL-C. Type 2 diabetes mellitus is regarded as a predictor of mortality from cancer including colon cancer, pancreatic cancer, lung and breast cancer in female, and liver and bladder cancer in male [36]. The increase in circulating levels of MMP-9 and ET-1 could be a possible linkage between MetS and cancers. It has been demonstrated that ET-1 and MMP-9 are both related to cancer development, progression and metastasis by suppressing apoptosis, and enhancing angiogenesis and mitosis [13, 14]. It has been demonstrated that ET-1 protects colon carcinoma cells from FasL-induced apoptosis [74] and prevent rat fibroblasts and human endothelial cells from serum-deprivation-induced apoptosis [13]. A transgenic animal study has demonstrated the involvement of MMP-9 in FasL-induced apoptosis by showing a significantly lower level of apoptosis in MMP-9 deficient mice [75]. In vitro studies revealed that ET-1 also serves as a mitogen in cell lines of colorectal cancer, ovarian cancer, prostate cancer, epithelial tumors, sarcoma and melanoma [13]. It has also been demonstrated in a co-culture in vitro study that ET-1 generated by human ovarian cancer cells can stimulate the carcinoma-associated fibroblasts [76]. Besides cancer cells, ET-1 also promotes the growth of endothelial cells and vascular smooth muscle cells, hence may facilitate angiogenesis in tumor [13]. It has been demonstrated that ET-1 is able to stimulate angiogenesis in a rat corneal model with same efficacy to vascular endothelial growth factor (VEGF) [77] and stimulate angiogenesis in subcutaneously implanted matrigel plugs in mice while co-treat with VEGF [78]. A study in 2008 has demonstrated the involvement of MMP-9 in activating VEGF and thus promotes angiogenesis [79]. Another study using transgenic zebra fish has demonstrated that ET-1 overexpression can lead to hepatocarcinagenesis [80]. It is reported that ET-1 can stimulate the expression of MMP-9, which contributes to the growth and metastasis of cancer [80]. The increase in circulating ET-1 and MMP-9 observed in the subjects with MetS and with certain metabolic abnormalities in the present study could be a possible explanation to the increased risk of having cancer in people with MetS while cancer patients with MetS might have a faster development of cancer due to the elevated circulating level of ET-1 and MMP-9.

In conclusion, the increase in circulating levels of ET-1 and MMP-9 has been observed in subjects with MetS and might be associated with the underlying causes of the increased risk of cardiovascular diseases, diabetes mellitus and cancer in people with MetS. Further research is needed to investigate the exact role of ET-1 and MMP-9 in the development of cancer, diabetes and cardiovascular disease in relation to metabolic syndrome.
